# Association of vertebral compression fractures with physical performance measures among community-dwelling Japanese women aged 40 years and older

**DOI:** 10.1186/s12891-017-1531-3

**Published:** 2017-04-28

**Authors:** Kazuhiko Arima, Yasuyo Abe, Takayuki Nishimura, Takuhiro Okabe, Yoshihito Tomita, Satoshi Mizukami, Mitsuo Kanagae, Kiyoshi Aoyagi

**Affiliations:** 1 Department of Public Health, Nagasaki University Graduate School of Biomedical Sciences, 1-12-4 Sakamoto, Nagasaki, 852-8523 Japan; 2 Department of Rehabilitation, Nishi-Isahaya Hospital, Isahaya, Japan

**Keywords:** Vertebral compression fractures, Physical performance, Walking speed, Chair stand time, Functional reach test

## Abstract

**Background:**

Numerous reported studies have shown that vertebral compression fractures are associated with impaired function or disability; however, few examined their association with objective measures of physical performance or functioning.

**Methods:**

We examined the association of vertebral compression fractures with physical performance measures in 556 Japanese women aged 40–89 years. Lateral spine radiographs were obtained and radiographic vertebral compression fractures were assessed by quantitative morphometry, defined as vertebral heights more than 3 SD below the normal mean. Measures of physical performance included walking speed, chair stand time and functional reach. Adjusted means of performance-based measures according to the number and severity of vertebral compression fractures were calculated using general linear modeling methods.

**Results:**

After adjusting for age, body mass index, back pain, number of painful joints, number of comorbidities and regular physical activities, the walking speed of women with two or more compression fractures (1.17 m/s) was significantly slower than that of women without compression fracture (1.24 m/s) (*p* = 0.03). Compared with women without compression fracture, chair stand time was longer in women with two or more compression fractures (*p* = 0.01), and functional reach was shorter (*p* = 0.01). No significant differences were observed in walking speed, chair stand time, or functional reach between women with one compression fracture and those without compression fracture.

**Conclusions:**

Having multiple vertebral compression fractures affects physical performance in community-dwelling Japanese women. Poor physical functioning may lead to functional dependence, accelerated bone loss, and increased risk for falls, injuries, and fractures. Preventing vertebral compression fracture is considered important for preserving the independence of older adults.

## Background

Osteoporosis has been characterized as a skeletal disorder of reduced bone strength that leads to an increased risk for fracture [1]. The estimated number of patients with lumbar spine (L2-L4) and femoral neck osteoporosis in Japan was approximately 6.4 and 11 million, respectively [2]. Vertebral compression fracture is the most prevalent manifestation of osteoporosis [3]. Vertebral fractures were reported to occur in approximately 20% of postmenopausal women in population-based studies [4–6], but two-thirds of vertebral fractures do not come to clinical attention [7, 8], because symptoms are absent or missed [9, 10]. In Japan, medical and care expenditures on spine fracture were estimated at 9,895 hundred million yen [11],

Physical performance measures have been applied in geriatric studies. Poor physical performance measures are associated with medical problems [12], disability in activities of daily living (ADL) [13, 14], and increased risk of falls [15–17]. Thus, physical performance measures capture information about these physical factors above. In addition, the elderly with poor physical performance have an increased risk of hip and non-spine fractures and lower bone mineral density [18–20].

Numerous studies showed that vertebral compression fractures are associated with back pain, impaired function or disability [21–29], most of which used subjective measures, not objective ones. A few published studies examined the association of the number of vertebral compression fractures with objective measures of physical performance and functioning [23, 25, 26, 30]. We examined the association of the number and severity of vertebral compression fractures with objective measures of physical performance among community-dwelling Japanese women.

## Methods

### Study design and participants

The subjects were Japanese women aged 40–89 years who participated in the Hizen-Oshima Study. Details of the Hizen-Oshima Study have been published previously [27, 31]. We recruited community-dwelling women aged 40 years and older in Oshima, Nagasaki prefecture, Japan. The women were identified by the municipal electoral list and were invited to participate through a single mailing. The town of Oshima has a population of approximately 5800; all women aged 40 and older (n >2000) were invited to participate. The examinations were performed at the Oshima Health Center between 1998 and 1999. A total of 586 women participated in the study. The mean age of participants (63.9 years) was significantly higher than that of non-participants (61.1 years). All participants were noninstitutionalized and living independently. All subjects gave written informed consent before enrolling. Recently, Cawthon et al. [30] reported that poorer physical performance was associated with an increased risk of incident radiographic vertebral fracture in men. We analyzed our baseline data of women in order to reinforce this evidence.

### Spine radiographs and vertebral compression fractures

Lateral radiographs were obtained with the subject lying in a lateral decubitus position with knees bent. All radiographs were obtained using a tube-to-film distance of 105 cm, with the tube positioned approximately over T-8 for thoracic films and L-2 for lumbar films. The anterior, medial, and posterior heights of each vertebral body (T-4 to L-4) were measured on the lateral films with the aid of a microcomputer-linked caliper. Vertebral heights were measured on the thoracic film for thoracic vertebrae, and on the lumbar film for lumbar vertebrae. The points indicating the border of the vertebral centrum were chosen based on the procedure described by Gallagher et al. [32] and Spencer et al. [33]. Radiographic images were evaluated by one orthopedic surgical specialist (KA). A vertebral compression fracture was defined as a vertebra with at least one dimension (anterior, medial, or posterior height) more than 3 standard deviations (SDs) below the vertebra-specific population mean on the radiographs [34]. For example, the means and SDs of the anterior, medial, or posterior heights of subjects’ T4 vertebrae were calculated. ([Mean anterior height of T4] - [3 × SD of anterior height of T4]; [mean medial height of T4] - [3 × SD of medial height of T4]; and [mean posterior height of T4 - [3 × SD of posterior height of T4]) were calculated. Each calculated value was compared with the actual anterior, medial, or posterior height. If at least one dimension (actual anterior, medial, or posterior height) was less than the calculated value, it was regarded as a T4 vertebral compression fracture. Furthermore, based on the extent of vertebral height reduction, mild compression fracture was recorded as 3–4 SDs, and severe compression fracture as >4 SDs below the normal mean value. Mild vertebral compression fracture was defined as at least one mild compression fracture without severe compression fractures (at least one mild but no severe compression fracture). Severe vertebral compression fracture was defined as one or more severe compression fractures (at least one severe compression fracture).

### Measurements

All participants were asked if they experienced back pain on most days during the previous month, and if they had any comorbidity, including cancer, heart disease, kidney disease, liver disease, diabetes mellitus, history of gastrectomy, thyroid disease, collagen disease, lung disease, stroke, or Parkinson’s disease. Information on regular physical activity was collected; participants were asked whether they do any physical activity (e.g., walking, jogging, or bicycling) long enough to work up a sweat at least once a week. Information on the number of painful non-spine joints was based on the subject’s response to the following question: “Which of your joints have ever been painful on most days during the previous month?” Specific response categories (shoulders, elbows, wrists, hands and fingers, hips, knees, ankles, and feet) for both sides of the body were provided on an illustration of the skeleton.

Measures of physical performance included walking speed (assessment of balance and lower extremity strength) [35], chair stand time (assessment of lower extremity strength) [36] and functional reach (assessment of balance and posture) [37], according to the previous report [19]. These performances are reported to be associated with balance, posture or lower limb strength [35–39]. Women who had difficulty performing the test safely, such as needing a walking aid, recent lower limb fracture or operation or neurological comorbidity, were not examined. Trained physical therapists conducted the performance tests. Walking speed was calculated as the time required for subjects to walk a 6-m course bounded by two lines at their usual pace (average of two trials). Chair stand time was measured as the time it took (average of two trials) to stand up from a standard chair five times; the subjects were asked not to use their arms for assistance. Functional reach was calculated as the difference between two measurements (average of three trials), as follows: the subjects first stood comfortably upright, facing forward, hand in a fist, with their arm extended next to a yardstick mounted on a wall. They then reached forward as far as possible without stepping or losing their balance. Height and weight were measured in light clothing and without shoes. Body mass index (BMI) was calculated as weight (kg)/height (m)^2^.

### Statistical analysis

Women with missing values for any variables were excluded from analysis, leaving 556 women for data analysis. Analysis of variance (ANOVA) and multiple comparisons using the Tukey method for continuous variables and Fisher’s exact test for categorical variables were used to determine the significance of differences among groups according to the number of vertebral compression fractures (no, one, and two or more). Age-specific means of physical performance measures were performed using ANOVA. Adjusted means of performance-based measures according to the number of vertebral compression fractures (no, one, and two or more) and the severity of vertebral compression fracture (no, at least one mild but no severe, and at least one severe) were calculated using general linear modeling methods (analysis of covariance). Age, BMI, back pain, number of non-spine painful joints, the number of comorbidities and regular physical activity was included simultaneously in the model as covariates. We examined the independent association of each variable above with physical performance measures. All statistical analyses were performed using SAS version 9.2 software (SAS Institute, Cary, NC, USA).

## Results

Characteristics of subjects are shown in Table 1. The mean age of subjects was 64.4 years old. Mean walking speed was 1.24 m/s, mean chair stand time was 9.4 s, and mean functional reach was 25.6 cm. A total of 30% of the subjects had back pain, and 52% engaged in regular physical activity; 14.2% (79) had one or more vertebral compression fractures; 6.9% (38) had one compression fracture; and 7.4% (41) had two or more compression fractures. Women with vertebral compression fractures were significantly older, shorter and lighter than those without vertebral compression fracture. Women with vertebral compression fractures had significantly poorer physical performance (slower walking speed, longer chair stand time and shorter functional reach) and more prevalent back pain compared with those without vertebral compression fracture.

**Table 1:** Basic characteristics of subjects according to compression fracture status (*n* = 556)

Variable	Total (*n* = 556)	No compression fracture (*n* = 477)	One compression fracture (*n* = 38)	Two or more compression fractures (*n* = 41)
	Mean (SD, 95% CI)			
Age (years)	64.4 (9.5, 63.6–65.2)	63.1 (9.3, 62.3–64.0)	69.5 (6.9, 67.2–71.8) ^a^	73.6 (7.1, 71.3–75.8) ^a^
Height (cm)	149.7 (6.1, 149.2–150.2)	150.5 (5.7, 150.0–151.0)	146.2 (5.5, 144.4–148.0) ^a^	143.6 (6.6, 141.6–145.7) ^a^
Weight (kg)	52.4 (8.7, 51.7–53.1)	53.1 (8.7, 52.3–53.9)	49.7 (7.6, 47.2–52.2) ^a^	46.8 (7.4, 44.5–49.2) ^a^
Body mass index (kg/m^2^)	23.3 (3.4, 23.1–23.6)	23.4 (3.5, 23.1–23.7)	23.2 (3.5, 22.1–24.4)	22.6 (2.7, 21.8–23.5)
Walking speed (m/s)	1.24 (0.25, 1.22–1.26)	1.26 (0.24, 1.24–1.28)	1.17 (0.28, 1.08–1.26)	1.05 (0.24, 0.97–1.13) ^a^
Chair stand time (s)	9.4 (3.1, 9.1–9.6)	9.1 (2.8, 8.8–9.3)	10.3 (2.9, 9.4–11.3) ^a^	11.8 (4.4, 10.4–13.2) ^a^
Functional reach (cm)	25.6 (7.2, 25.0–26.2)	26.3 (7.0, 25.7–27.0)	22.7 (6.8, 20.5–25.0) ^a^	19.7 (6.8, 17.6–21.9) ^a^
	Number (%)			
Back pain	164 (29.5)	128 (26.8)	18 (47.4) ^b^	18 (43.9) ^b^
Regular physical activity	288 (51.8)	248 (52.0)	23 (60.5)	17 (41.5)
Postmenopausal women	510 (91.7)	431 (90.4)	38 (100.0) ^b^	41 (100.0) ^b^
	Median (1st, 3rd quartile)			
No. of non-spine painful joints	0 (0, 2)	0 (0, 2)	0.5 (0, 2)	0 (0, 2)
No. of comorbidity	0 (0, 1)	0 (0, 1)	0 (0, 1)	0 (0, 1)

^a^ *P* < 0.05 compared with no compression fracture (ANOVA & Tukey method)

^b^ *P* < 0.05 compared with no compression fracture (Fisher’s exact test)

Table 2 shows mean and standard deviation (SD) of vertebral height, and %height reduction values [(mean-vertebral height)/mean] corresponding to mild (mean-3SD) and severe (mean-4SD) vertebral fracture cutoffs for each vertebral dimension. Percent height reduction values corresponding to mild vertebral fracture cutoffs were between 20.3 and 38.3%, and those corresponding to severe vertebral fracture cutoffs were between 27.0 and 51.0%.

**Table 2:** Mean and standard deviation (SD) of vertebral height (mm) and % reduction values [(mean-vertebral height)/mean]

	mean	SD	Mild % reduction: 100*[1 - (mean-3SD)/mean] (%)	Severe % reduction: 100*[1 - (mean-4SD)/mean] (%)
T4A	18.75	1.40	22.4	29.8
T5A	19.10	1.50	23.6	31.5
T6A	19.13	1.87	29.3	39.1
T7A	19.43	1.90	29.4	39.2
T8A	19.98	2.19	33.0	43.9
T9A	20.80	2.11	30.5	40.7
T10A	21.67	1.81	25.1	33.4
T11A	22.30	2.10	28.3	37.7
T12A	23.90	3.05	38.3	51.0
L1A	26.19	3.07	35.2	46.9
L2A	27.66	2.95	32.0	42.7
L3A	28.77	2.49	26.0	34.6
L4A	28.32	2.48	26.2	35.0
T4M	18.87	1.44	22.9	30.5
T5M	19.33	1.44	22.4	29.8
T6M	19.63	1.66	25.4	33.8
T7M	19.88	1.83	27.6	36.8
T8M	20.19	2.02	30.1	40.1
T9M	20.81	1.93	27.9	37.2
T10M	21.70	1.83	25.3	33.8
T11M	22.90	1.92	25.1	33.4
T12M	24.61	3.00	36.6	48.7
L1M	26.52	3.29	37.2	49.7
L2M	27.35	2.96	32.5	43.3
L3M	27.94	2.53	27.2	36.3
L4M	27.57	2.80	30.5	40.6
T4P	20.57	1.68	24.6	32.8
T5P	21.26	1.60	22.6	30.1
T6P	21.83	1.57	21.6	28.8
T7P	22.21	1.68	22.7	30.2
T8P	22.25	1.70	22.9	30.5
T9P	22.37	1.69	22.7	30.2
T10P	23.44	1.68	21.5	28.6
T11P	25.19	1.95	23.2	31.0
T12P	27.67	2.18	23.7	31.6
L1P	29.72	2.19	22.1	29.4
L2P	30.09	2.03	20.3	27.0
L3P	29.70	2.02	20.4	27.2
L4P	27.95	2.22	23.9	31.8

Physical performance decreased with age (Table 3); walking speed and functional reach decreased, and chair stand time increased (longer time required to complete the test represents a decline in function).

**Table 3:** Means (SD) of physical performance measures by age group

Age group (years)	40–49	50–59	60–69	70–79	80-	*P* for trend
	(*n* = 41)	(*n* = 118)	(*n* = 211)	(*n* = 161)	(*n* = 25)	
Walking speed (m/s)	1.44 (0.20)	1.34 (0.19)	1.29 (0.22)	1.10 (0.22)	0.93 (0.24)	<0.0001
Chair stand time (s)	6.7 (1.2)	8.1 (1.7)	9.1 (2.3)	10.7 (3.1)	13.4 (6.2)	<0.0001
Functional reach (cm)	33.0 (5.9)	29.8 (6.1)	25.0 (6.3)	22.6 (6.4)	18.2 (6.4)	<0.0001

Table 4 shows the adjusted means of physical performance measurements according to the number of vertebral compression fractures. After adjusting for age, BMI, back pain, number of non-spine painful joints, the number of comorbidities and regular physical activity, the walking speed of women with two or more compression fractures (1.17 m/s) was significantly slower than that of women without compression fracture (1.24 m/s) (*p* = 0.03). Compared with women without compression fracture, chair stand time was longer in women with two or more compression fractures (10.4 s vs. 9.3 s) (*p* = 0.01), and functional reach was shorter (23.2 cm vs. 25.9 cm) (*p* = 0.01). No significant differences were observed in walking speed, chair stand time, or functional reach between subjects with one compression fracture and those without compression fracture. In a multivariate model, slower walking speed was associated with older age, number of non-spine painful joints, number of comorbidities and lack of regular physical activity. Longer chair stand time was associated with older age, number of non-spine painful joints and lack of regular physical activity. Shorter functional reach was associated with older age. Additionally, we did the same analysis including upper or lower extremity joint pain instead of non-spinal painful joints. Slower walking speed was independently associated with number of upper and lower extremity joint pain, and chair stand time with lower extremity joint pain.

**Table 4:** Multivariate-adjusted mean (standard error) of walking speed, chair stand time and functional reach according to number of vertebral compression fractures

	Vertebral compression fracture	Adjusted mean (SE)	*P* value
Walking speed (m/s)	No	1.24 (0.01)	
	One	1.23 (0.03)	0.68
	Two or more	1.17 (0.03)	0.03 ^a^
Adjusted factors		Estimate (SE)	
Age (year)	+1 year	−0.012 (0.001)	<0.001
BMI	+1 kg/m^2^	−0.003 (0.003)	0.22
Back pain	1, yes; 0, no	−0.023 (0.020)	0.24
No. of non-spine painful joints	+1	−0.018 (0.004)	<0.001
No. of comorbidity	+1	−0.039 (0.014)	0.005
Regular physical activity	1, yes; 0, no	0.041 (0.017)	0.02
Chair stand time (s)	No	9.3 (0.1)	
	One	9.6 (0.4)	0.41
	Two or more	10.4 (0.4)	0.01 ^a^
Adjusted factors			
Age (year)	+1 year	0.14 (0.13)	<0.001
BMI	+1 kg/m^2^	−0.004 (0.03)	0.91
Back pain	1, yes; 0, no	0.04 (0.25)	0.87
No. of non-spine painful joints	+1	0.16 (0.05)	0.003
No. of comorbidity	+1	0.25 (0.18)	0.16
Regular physical activity	1, yes; 0, no	−0.70 (0.22)	0.002
Functional reach (cm)	No	25.9 (0.3)	
	One	24.7 (1.0)	0.28
	Two or more	23.2 (1.0)	0.01 ^a^
Adjusted factors			
Age (year)	+1 year	−0.37 (0.03)	<0.001
BMI	+1 kg/m^2^	−0.12 (0.08)	0.12
Back pain	1, yes; 0, no	−0.85 (0.60)	0.16
No. of non-spine painful joints	+1	0.08 (0.13)	0.52
No. of comorbidity	+1	−0.04 (0.42)	0.92
Regular physical activity	1, yes; 0, no	0.17 (0.53)	0.75

*BMI* Body mass index

^a^ compared with women without compression fracture

We also compared multivariate-adjusted means of physical performance measures according to severity of vertebral compression fracture. Women with severe vertebral compression fractures (at least one severe compression fracture) had significantly slower walking speed (*p* = 0.03) and longer chair stand time (*p* = 0.01) compared with women without compression fracture (data not shown). No significant differences were observed in walking speed and chair stand time between women with mild compression fracture (at least one mild but no severe compression fracture) and those without. Functional reach tended to be shorter in those with mild compression fracture (*p* = 0.06) and severe compression fracture (*p* = 0.08) than in those with no compression fracture. Women with severe vertebral compression fractures tended to have multiple vertebral compression fractures; median number (range) of vertebral compression fractures was 1 (1–3) in women with mild vertebral compression fracture and 2 (1–12) in those with severe vertebral compression fracture.

We examined multivariate-adjusted means of physical performance measures according to location of vertebral compression fracture (thoracic vs. lumbar). Longer chair stand time was associated with one thoracic compression fracture. Shorter functional reach was associated with two or more thoracic compression fracture.

## Discussion

We showed that two or more vertebral compression fractures were associated with poor physical performance in walking speed, chair stand time, and functional reach. Huang et al. [25] reported that the number of recent vertebral fractures was associated with reduced functional reach and walking speed. Pluijm et al. [26] reported that the number of vertebral deformities was significantly associated with poor performance (cardigan test, walking test and chair stand test). These findings suggest that vertebral compression fracture affects various physical performance measures.

Previous studies have shown the relationship between vertebral compression fractures and impaired function (difficulty with activities of daily living [ADL]) or disability [21–29]. Poor physical performance test results (decreased functional reach and walking speed, and increased chair stand time) are reported to be predictors of impaired function or disability [13, 40, 41]. Vertebral compression fractures are likely to cause functional limitations, such as difficulty with ADL and physical performance.

Abnormal trunk posture resulting from vertebral compression fractures has been reported to be associated with functional disabilities [42, 43]. Antonelli-Incalzi et al. [44] showed that longer occiput-wall distance, which indirectly evaluates thoracic kyphosis due to vertebral compression fractures [45], was associated with slower walking speed, reduced balance, and longer chair stand time in elderly women. Hirose et al. [46] showed that abnormal posture of the trunk (thoracic and lumbar kyphosis) was associated with slower walking speed and shorter functional reach among the community-dwelling elderly population. In our study, longer chair stand time was associated with one thoracic compression fracture, and shorter functional reach was associated with two or more thoracic compression fracture.

We previously reported that spinal posture in forward inclination was associated with impaired physical performance in 6-m walking time, chair stand time, and functional reach [39]. Lyles et al. [23] postulated that osteoporotic compression fractures affect the alignment of the entire trunk, leading to a reduction in motion and strength. The shift forward in the center of gravity that occurs with progressive kyphosis from vertebral compression fracture could make trunk movements in a forward direction more unsteady and limited.

Some vertebral compression fractures are associated with increased risk of back pain [22, 25, 47]. In situations in which both vertebral compression fracture and back pain are present, functional losses could occur from the symptoms caused by the compression fracture and/or from the alteration in trunk alignment [29]. In this study, having vertebral compression fractures was significantly associated with poor physical performance independent of back pain and other covariates. These results possibly mean vertebral compression fractures affect physical performances via abnormal trunk posture.

Comorbidities are associated with decreased physical function, and the more the number of comorbidities is, the poorer the physical function is [48]. Thus, we included the number of comorbidities in the model as a covariate.

Our definition of vertebral compression fracture used the vertebral height and its SD, according to Ross et al. [34]; they reported that the cutoff of 3 SD rarely misclassified normal vertebrae as fractured (specificity = 99.9%). However, this cutoff correctly identified about 70% of the incident fractures. A less stringent criterion (2 SD below the mean: specificity = 97.7%) identified about 85–90% of true fractures. Thus, our results may have underestimated the prevalence of compression fractures, which may contribute to Type II error of the association.

The study has several limitations. First, because this was a cross-sectional design, a causal relationship was not necessarily shown by our results. Longitudinal studies are required to clarify the relationships between vertebral compression fracture and physical performance. Second, the subjects were community-dwelling women who voluntarily participated; women with poorer physical performance might not have participated in the present study, which might have affected the results. Third, there are no results of the Timed Up & Go Test of physical performance in our analysis. Fourth, the present study included only Japanese women; therefore, it may be not possible to extrapolate the results to men or people of other ethnicities. Nevertheless, the present data clearly showed an association of vertebral compression fracture with deterioration of physical performance, independent of age and other covariates.

## Conclusion

Our results suggest that multiple vertebral compression fractures affect physical performance (slower walking speed, lower chair stand time and shorter functional reach) in community-dwelling Japanese women, independent of age and other covariates. Because poor physical functioning may lead to functional dependence, accelerated bone loss, and increased risk for falls, injuries (e.g., sprain, abrasion, laceration or dislocation after falling), and fractures [25], preventing vertebral compression fracture is important for the preservation of older adults’ independence.

## Abbreviations

ADL: Activities of daily living
BMI: Body mass index
